# Hæmaturia in the Pre-Eruptive Stage of Measles

**Published:** 1931

**Authors:** F. G. Jenkins


### HÆMATURIA IN THE PRE-ERUPTIVE STAGE OF MEASLES.

BY

F. G. JENKINS, M.B., CH.B.

THE occurrence of a copious hæmaturia during the prodromal stage of measles is sufficiently rare to bc recorded.

On the 1st January, 1931, a boy, six years of age, became ill with sneezing, indefinite malaise and slight backache. There had been no previous illness. On the evening of the second day he passed, per urethram, without pain, what was thought to be pure blood. I saw him at 8.30 p.m. and found him in bed with a temperature of 101·4° F. and a pulse-rate of 112. The pharynx and nose were congested, but the retinæ, skin, lungs, heart, abdomen and limbs revealed nothing abnormal. The spleen was not felt, and there was no trace of œdema. About 10 fluid ounces of bright red, hæmorrhagic urine lay in a receptacle by the bedside, and on microscopical examination, without centrifugalizing, this was found to be full of erythrocytes.

On the 3rd January the temperature sank to 98° F. The urine was still full of blood. During the 4th and 5th January the temperature rose steadily to 101° F. ; a cough developed, and there was slight photophobia, but there was much less hæmaturia. After my visit on the 5th January, the fourth day of the illness, the parent observed a rash appear on the forehead and spread rapidly downward, so that by the following afternoon, when I saw the child again, the face, trunk and limbs were covered by a morbilliform rash. Koplik's spots were not found, but they had had time to fade during the eighteen hours the rash had been out. The rash was not unduly hæmorrhagic and was typical. The rapid development of respiratory signs confirmed the diagnosis of measles. By the 11th January the exanthem had faded, leaving the characteristic faint brown staining and all trace of blood had gone from the urine. A mild broncho-pneumonia kept up the temperature, but convalescence was established by the 20th January. At no time was the spleen felt, and there were no other hæmorrhages.

About ten days after the onset a younger brother, aged eighteen months, developed typical measles. There were numerous other cases in the neighbourhood.

On the 3rd February, 1931, Dr. J. A. Birrell, Hon. Physician to the Bristol Royal Hospital for Sick Children and Women, was kind enough to take the boy into hospital for investigation. He reported that radiographs showed no trace of calculus; repeated urinary examinations could detect neither albumen nor blood; and the urea concentration test gave percentages of 2·2 and 2·8. The cardio-vascular system was normal.

On the 10th December, 1931, I found that the boy had been in good health throughout the year. The urine and its deposit showed no abnormality.

It is evident that this was not a case of hæmorrhagic measles, as the hæmorrhage was confined to one system only, and occurred only in the prodromal period. The hospital investigation and the absence of other clinical signs of nephritis and calculus, at the time of the illness, rule out these conditions as a cause of the hæmaturia. A careful search through the available literature[1] failed to discover a similar case. John Ruhräh notes: "Epistaxis and other hæmorrhages may occur, especially on the third or fourth day"[2] (of the prodromal period); whereas Dawson Williams, describing the same period, says: "In some epidemics, epistaxis has been observed in a large proportion of cases, occasionally to a serious extent."[3] I think that the hæmaturia in this case must be a hæmorrhage of the same type as the epistaxis noted by Ruhräh and Williams, that is, either toxic or congestive in origin.

REFERENCES.

[1] *Index Medicus*, 1912 to 1931; *Medical Annual*, 1887 to 1931; Nothnagel's *Encyclopedia of Practical Medicine*; Pepper's *System of Practical Medicine*, 1885, vol. i.; Ker's *Infectious Diseases*, revised by Claude Rundle; *Infectious Diseases*, by E. W. Goodall, Third Edition; *Acute Infectious Diseases* by Rolleston, Second Edition.

[2] *System of Medicine*, Osler and Macrae, 1907, vol. ii. ("Measles" by John Ruhräh), p. 381.

[3] *System of Medicine*, Allbutt and Rolleston, 1906, vol. ii., part 2 ("Measles" by Dawson Williams), p. 389.

